# Supplementary material for: COVID-19 CG: Tracking SARS-CoV-2 mutations by locations and dates of interest
Source: bioRxiv. 2020 Sep 28:2020.09.23.310565. Preprint. [Version 2] doi: 10.1101/2020.09.23.310565 (PMC7523124; doi:10.1101/2020.09.23.310565)
Supplement: 1 [file NIHPP2020.09.23.310565-supplement-1.pdf]

bioRxiv preprint doi: https://doi.org/10.1101/2020.09.23.310565. this version posted September 28, 2020. The copyright holder for this preprint (which was not certified by peer review) is the author/funder. It is made available under a CC-BY 4.0 International license.

Supplemental Figures

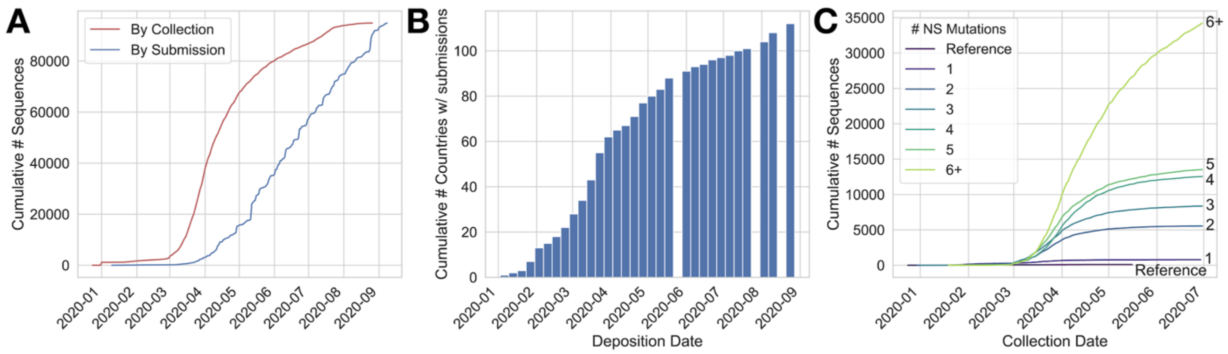

Figure S1. The number of global SARS-CoV-2 genome sequences and mutations is accumulating. Data shown as of September 9, 2020. **(A)** Sequence deposition in GISAID continues at a steady pace, albeit there is a lag between collection (red line) and submission date (blue line). The rate of sequence submission is steady at >10,000 genomes per month. **(B)** More than 100 countries have deposited SARS-CoV-2 genomes in GISAID. **(C)** The number of SARS-CoV-2 variants with more than six nonsynonymous (NS) mutations continues to increase.

bioRxiv preprint doi: https://doi.org/10.1101/2020.09.23.310565. this version posted September 28, 2020. The copyright holder for this preprint (which was not certified by peer review) is the author/funder. It is made available under a CC-BY 4.0 International license.

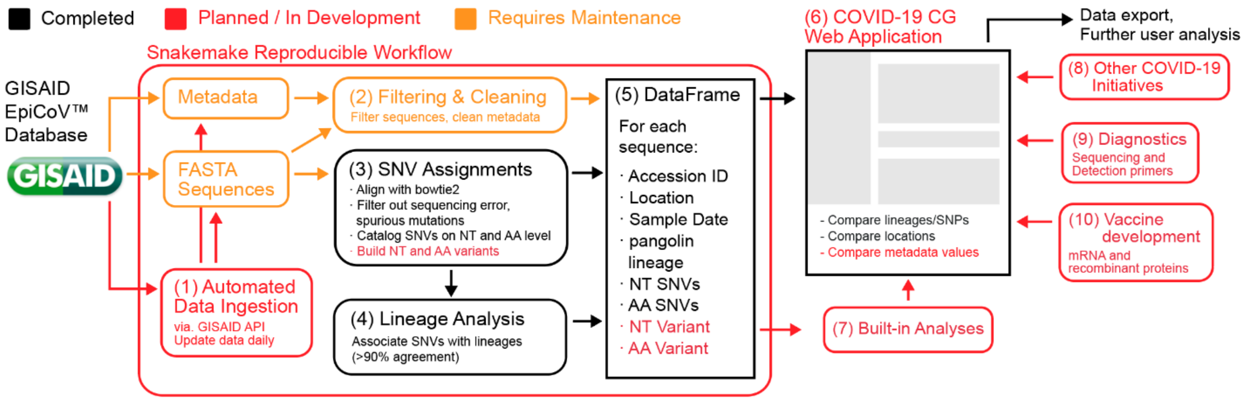

Figure S2. COVID-19 CG computational workflow. **(1)** Starting from the GISAID database, sequences are continuously updated, manually for now, but ultimately via automated data ingestion. **(2)** Based on best practices, we filter out sequences on NextStrain's exclusion list, non-human isolates, <29,700 nt, or with >5% ambiguous base calls (van Dorp et al., 2020). **(3)** SNVs at the nucleotide and amino acid level are determined by aligning (via *bowtie2*) each sequence to the WIV04 reference, a high quality December, 2019 isolate recommended by GISAID; NextStrain uses the 100% identical Wuhan-Hu-1 (Langmead et al., 2009). Importantly, spurious SNVs and probable sequencing errors are filtered out prior to downstream analysis. **(4)** Viral lineages, defined by the *pangolin* tool, are provided by GISAID. In accordance with *pangolin*, SNVs present in >90% of sequences within each lineage are assigned as lineage-defining SNVs. **(5)** The curated data and metadata, SNVs, and lineage-assigned SNVs are associated with their respective sequence identifier and compiled into a compact data set. **(6)** These data are uploaded onto the COVID-19 CG web application. **(7)** New analyses will be built into the COVID-19 CG application throughout the course of the pandemic. **(8-10)** Features and modules that integrate knowledge from other COVID-19 initiatives are continuously incorporated into COVID-19 CG.
